# Supplementary material for: Are carbon emissions trading and green financial instruments synergistic? -Comprehensive quantitative research based on content analysis
Source: PLoS One. 2024 Mar 7;19(3):e0298601. doi: 10.1371/journal.pone.0298601 (PMC10919601; doi:10.1371/journal.pone.0298601)
Supplement: S1 Data — (ZIP) [file pone.0298601.s001.zip › Data/地级市Z_F工作报告.zip!/║■─╧/╓Ω╓▐╩╨/└┤╫╘╣1⁄2╓┌║┼í╛┬φ┐╦╩2╛▌═°í┐_2015.docx]

各位代表：

现在，我代表市人民政府向大会报告工作，请予审议，并请各位政协委员和其他列席人员提出意见。

一、2014年工作回顾

过去的一年，面对经济下行的较大压力和改革发展稳定的繁重任务，在省委、省政府和市委的坚强领导下，我们紧密团结和依靠全市人民，积极应对经济发展新常态，保持定力，精准发力，全力以赴稳增长、促改革、调结构、惠民生，经济发展稳中有进、进中见好，社会大局和谐稳定。

（一）在努力克服经济下行压力中保持了经济平稳较快发展

经济发展迈上新台阶。全年GDP突破2000亿元，达到2160亿元，增长10.5%；规模工业增加值突破1000亿元，达到1013亿元，增长11.6 %；完成公共财政收入264亿元，增长12.1%；完成固定资产投资1837亿元，增长22%；实现社会消费品零售总额743亿元，增长12.6%；城镇居民人均可支配收入达到31453元，增长9.6%；农民人均纯收入达到14430元，增长11.8 %。

（二）在强力推进结构调整中加快了产业转型升级步伐

工业经济提质增效。围绕清水塘老工业基地搬迁改造、湘江保护和治理省政府“一号重点工程”、醴陵烟花鞭炮产业整合、攸县煤矿关停并转，坚决淘汰了一批企业。大力实施工业振兴行动。全力推进“中国动力谷”建设，获批国家轨道交通装备产业区域集聚发展试点市。轨道交通、汽车、航空产业产值分别增长23%、16%、13.3%。实施国家、省级科技项目突破100项，自主设计建造的国内首条、世界第二条8英寸IGBT专业芯片生产线正式投产，自主研发了全球首台储能式低地板有轨电车等新产品，完成高新技术产业增加值466亿元，增长17.7%。大力推进企业成长“十百千”工程，突出园区发展，园区技工贸收入达2284亿元，增长22.3%。株洲高新区跻身国家级高新区前40位，前移6位。非公经济发展进一步加快，非公经济增加值1242亿元，占GDP比重达到57.5%。

现代服务业加快发展。推动服务业全面升级，完成第三产业增加值709亿元，增长11.2%。电子商务蓬勃发展，阿里巴巴·株洲产业带建成投入运营。现代物流不断壮大，南车物流、大美物流等项目建设稳步推进。文化旅游业发展持续升温，实现旅游业收入202亿元，增长18.1%。炎陵县被列入全省第二批文化旅游特色产业县。

现代农业稳步发展。实现农业增加值170亿元，增长4.5%。粮食种植面积400万亩，粮食总产量182万吨，实现“十一连丰”。经营规模化水平进一步提高，生猪规模化养殖比重达71%，提质增效蔬菜基地5600亩，农村土地流转率39.1%，高于全省平均水平10个百分点。生产专业化程度进一步提升，新增家庭农场236家、农民专业合作社517家，农产品标准化生产基地220万亩。水稻生产综合机械化水平达69%。经营品牌化效应进一步放大，新增国家级省级龙头企业21家、“三品一标”农产品认证52个、放心粮油示范企业（网点）55个。服务社会化支撑进一步强化，农技、气象、水文等产前、产中、产后社会化服务水平全面提升。

（三）在着力推进项目建设中夯实了发展基础

项目引进成效明显。坚持在国家和省级重大工作部署中捕捉机遇，一批重大项目在我市布局。成功获批国家自主创新示范区、国家新型城镇化综合试点城市、国家电子商务示范城市。加大招商引资力度，成功引进大汉惠普软件（湖南）信息产业园、台湾元创等一批重大项目。推进146个历年重点招商项目落地，履约率达72.6%。承接产业转移项目219个，实际到位资金169.2亿元，增长18.1%。

项目建设扎实推进。全力推进“项目建设年”活动。实现52个重点前期项目开工建设，重点建设项目完成投资336亿元。微软湖南创新中心正式揭牌，北汽二工厂、湖南云龙国际信息产业园、湖南移动数据中心、中国动力谷自主创新园、新芦淞白关（国际）服饰产业园、中华茶祖园、陶瓷艺术城等项目正在抓紧建设。醴陵旗滨玻璃、中航航空发动机维修中心、旭日陶瓷等一批项目建成投产，为产业加速发展增添了后劲。

要素保障支撑有力。全力破解土地、资金等要素瓶颈，大力开展“征拆征收攻坚行动”，征地拆迁交地3.2万亩、国有土地上房屋征收36.5万平方米，分别增长37%、28%，有力保障了项目建设的需求。积极推动政银企合作，对接签约项目73个，总金额315亿元。不断拓宽融资渠道，新增直接融资136亿元，增长25%。煤电油气等其他要素保障有力。

（四）在全面推进改革开放中激发了发展动力

各项改革释放新动力。深入开展“改革创新年”活动，围绕市委部署的7大类27项改革任务，以政府自身改革引领其他各项改革。推进行政审批制度改革，加大简政放权力度，市本级行政审批项目由302项精简至122项，精简率为60%。绿色通道、容缺受理、全程代办等特色服务获得李克强总理肯定。推进商事制度改革，全市新增私营企业6103家，增长150%。推进投融资体制改革，实施政府投资项目管理办法，严格规范政府投资行为。出台鼓励PPP模式投资的实施意见，向社会发布第一批项目38个。推进国企改革，完成了10家国有工业企业改革，央企“三供一业”改革经验全省推广。推进财税金融体制改革，预算公开试点范围扩大到所有县市区，财政专项资金管理逐步规范。“营改增”试点范围不断扩大。醴陵、攸县农商行正式挂牌营业。推进土地制度改革，荷塘区、芦淞区3.75万宗农村宅基地登记发证工作基本完成，茶陵县严塘镇及其他18个村土地承包经营权登记颁证试点顺利推进。推进两型改革，全面实行居民生活用电、用气、用水阶梯价格制度。获批“全国水生态文明城市试点”和“全国节水型社会建设示范区”。率先在全省推进大环保体制建设。建立企业环保及诚信信息公开制度和两型监测评价体系。同时，政府机构改革和事业单位分类改革、医药卫生体制改革、公共资源交易管理体制改革等有序推进。

开放合作迈出新步伐。积极实施“走出去”战略，大力拓展新兴市场，全年完成外贸进出口总额28亿美元，增长9.3%。全年实际利用外资8.24亿美元，增长18.9%，内联引资262亿元，增长14%。加快培育外向型企业，全市56家企业取得外贸业绩零突破，35家企业实现外贸业绩倍增。时代新材成功收购德国博戈公司。轨道交通装备出口到土耳其、南非、阿根廷等多个国家和地区。有色金属新材料产业出口基地创建为“全国外贸转型升级示范基地”。把株洲放在国家“三大战略”和湖南“一部一带”定位的大格局中去谋划和对接，在基础设施互联互通、承接产业转移、旅游、教育、卫生等方面开展了深入的交流和对接工作。湘赣开放合作试验区全面启动。荣获“中国最佳营商环境城市”称号。

（五）在大力推进城镇扩容提质中加快了城乡统筹发展

城市管理水平持续升级。全力冲刺全国文明城市创建。围绕美化、绿化、亮化、数字化“四化”工程，实施市容环境整治“百日攻坚战”，开展市容市貌、交通秩序、环境卫生、路灯亮化等十大专项整治行动，成效明显。强化城市管理考核，实行环卫行业“末位淘汰”。不断完善大城管格局，城市管理与体制创新荣获“中国人居环境范例奖”。

城乡建设和环境全面提质。着力推进“两供两治”建设，新建城市供水管网68公里，新建改建市政燃气管网79公里。白石港水质净化中心、龙泉污水处理厂三期工程竣工投产。霞湾、董家塅等污水处理厂提标改造工程开工。南郊垃圾场封场，城市生活垃圾焚烧发电厂投产运行。启动“光纤到户”工程。建立地下管线信息公共服务平台，实现了城区地下管线 “数字化”。全面落实湘江保护和治理省政府“一号重点工程”，大力实施“三个基本行动”，淘汰关停高能耗、高污染企业（生产线）98家（条），拆除烟囱49根，截流湘江株洲段沿岸排污口9个，湘江沿岸1公里内养殖场退出272家。清水塘老工业基地搬迁改造取得重要进展，旗滨玻璃株洲城区生产基地绿色搬迁全面完成，淘汰株冶10万吨炼铅生产线。湘江株洲段、洣水、渌水水质保持国家三类标准。市区环境空气质量良好天数达到234天，比上年增加20天。对30座重点小二型病险水库进行除险加固，建设“五小”水利工程2029处，新解决16.38万农村人口饮水不安全问题。美丽乡村建设有力推进。成功创建“国家森林城市”。

城镇扩容稳步推进。围绕打造“湖南东大门”、建设株醴都市区，实施“一轴一带”发展战略，编制完成市域城镇布局规划、东部新城及天易科技城概念规划、五县（市）县城总体规划修改和乡镇总体规划。坚持交通先行。全年完成交通建设投资113亿元。衡茶吉铁路、沪昆高铁株洲段贯通，荷塘大道、铜霞路二期竣工通车，建成S205炎陵沔渡至船形等干线公路。长株潭城际铁路、莲株公路改扩建工程、湘江六桥、湘江七桥、湘江大道、中环大道贯通工程等项目建设进展顺利。建成国省干线公路101公里，续建53公里，新开工建设118公里。建成农村公路360公里、改造危桥60座。醴娄高速纳入国网，东城大道、长株攸公路等项目前期工作扎实推进。大力发展公共交通，开通“定制公交”线路62条。

（六）在切实保障和改善民生中促进了社会和谐

社会保障不断强化。持续加大民生投入，涉及民生的财政支出208亿元，占公共财政预算支出的70.8%。“民生100”工程顺利完成。全市城镇新增就业6.1万人，新增农村劳动力转移就业1.7万人，失业人员再就业2.5万人，零就业家庭动态援助率保持100%。继续扩大社会保险覆盖面，新增企业养老保险参保4.7万人。为市区环卫工人买五险、供中餐、加工资、配廉租房。城区城镇居民和农村居民最低生活保障标准分别增加到420元和180元。1.8万农村五保对象住院基本医疗费用全额减免。开工建设保障性住房和各类棚户区改造1.84万套，开工率100%。开工建设安置房5482套，竣工封顶8109套。推进老旧小区物业管理试点工作，解决了城区1万多户居民房屋和6000多户土地登记发证历史遗留问题。积极推进精准扶贫，帮助3.14万困难群众实现脱贫。

社会事业全面发展。教育事业扎实推进。新增普惠性幼儿园102所，建设义务教育合格学校30所。市二中整体搬迁，原址转办公办初中。职教科技园5所院校建成开学。天元区、芦淞区、荷塘区获得全省首批“教育强区”称号。医药卫生事业加快发展。在全省率先取消“以药补医”。市妇幼保健院搬迁及改扩建稳步推进。临床重点专科建设三年行动计划开局良好，5个重点专科建设取得新突破。人口计生工作蝉联“省人口计生模范市”。文体事业不断发展。全面修复秋瑾故居。成功举办甲午年炎帝陵祭祖大典、全国第二届民盟教育论坛。省十二运会成绩位居全省前列。全面完成第三次经济普查登记和数据汇总工作。

社会大局和谐稳定。加强基层政权建设，顺利完成第九次村委会换届选举工作。深入推进平安株洲、法治株洲建设，全面建设立体化防控体系，狠抓治安源头治理，积极化解信访积案，妥善处理各类纠纷，综治工作成效明显。全面开展安全生产大检查，认真落实各项安全生产责任，安全生产形势平稳可控，隐患自查自改月报工作经验全省推广。大力开展食品药品安全专项整治行动，人民群众饮食用药安全得到有效保障。巩固和发展民族团结进步事业，成为全省唯一连续6次荣获“全国民族团结进步模范集体”称号城市。深入开展军民共建，“株洲舰”正式入列，扎实推进“全国双拥模范城市”六连冠工作，国防动员工作不断加强。驻株解放军、武警部队和民兵预备役，为全市经济社会发展作出了积极贡献。

与此同时，妇女儿童、老龄、残联、机构编制、外事侨务、宗教、广播电视、文化执法、国家安全、质监、海关、检验检疫、供销、人防、档案、市志、社科联、红十字、对口支援、移民、志愿者服务等工作都有新进步。

（七）在深入开展群众路线教育实践活动中改进了工作作风

结合“四型”政府建设和“环境优化年”活动，深入开展党的群众路线教育实践活动，铁腕整治“四风”问题，着力开展正风肃纪、提高机关办事效率等五个专项行动，切实解决困难群体保障、就业创业、看病贵、上学难、住房保障等九类突出问题，让群众看得见、感受得到作风建设成效，机关作风满意度民调测评排名全省第一。大幅压缩“三公经费”，全面停止新建楼堂馆所。全年精简会议和文件分别达28.6%、23.4%，清理调整单位办公用房近20万平方米，精简市级议事协调机构305个。认真处理市长热线和市长信箱中的群众咨询、建议和投诉共7万件，办结率为99.5 %。推行企业评议经济发展环境等举措，行政效能进一步提升。自觉接受市人大及其常委会法律监督、工作监督和市政协民主监督，充分吸纳各民主党派、工商联、无党派人士和社会各界的意见、建议。积极办理人大代表建议290件、政协委员提案490件，见面率、办结率、满意率均为100%。严格执行党风廉政建设责任制和廉洁从政有关规定，行政监察和审计监督不断加强。

各位代表！过去的一年，发展的成绩来之不易，积累的经验弥足珍贵。这是省委、省政府和市委坚强领导的结果，是市人大、市政协监督支持的结果，是全市人民团结拼搏、不懈努力的结果。在此，我代表市人民政府，向全市广大工人、农民、干部、知识分子和企业家，向各民主党派、工商联、无党派人士、各人民团体，向离退休老领导、老同志，向驻株部队、武警官兵、公安政法干警，向所有关心支持株洲发展的社会各界朋友，表示崇高的敬意和衷心的感谢！

在总结成绩的同时，我们也清醒地看到，株洲经济社会发展还存在一些亟待解决的困难和问题：一是实现经济平稳增长的任务艰巨。市场需求不足，部分企业经营困难，大项目、好项目不多的问题还比较突出。二是推动转型升级压力较大。清水塘老工业基地搬迁改造、湘江污染治理等工作难度大，新兴产业替代传统产业支撑发展的作用还不强。三是经济运行风险犹存。房地产、金融和地方性债务等方面长期叠加的风险有待化解。四是一些基层基础工作还有薄弱环节。安全生产形势不容乐观，改善民生和维护社会稳定还有大量工作要做。五是政府自身建设需要进一步加强。部分政府工作人员服务意识不强，办事效率不高，“为官不为”等问题还一定程度存在。对此，我们将采取有效措施切实加以解决。

二、2015年宏观形势和总体思路

2015年，是实施“十二五”规划的收官之年，也是做好“十三五”规划的谋划之年，更是全面深化改革、加快转型升级的关键之年。

2015年，世界经济形势复杂多变，国内经济已进入“中高速、优结构、转动力、多挑战”的新常态。对我市来说，新常态有新挑战：一是经济下行压力增大与扩大经济总量、提升发展质量之间的矛盾，需要我们找准切入点。二是传统产业急需转型与新兴产业成长缓慢的矛盾，需要我们找准增长点。三是资金、土地等要素外部约束加大与内部需求增加的矛盾，需要我们找准突破点。新常态有新机遇：一是改革红利不断释放。行政审批、投融资、土地制度、财税制度、户籍制度等方面的改革会不断地激发市场活力。二是政策效应逐渐显现。为了稳增长、调结构，中央和省里相继出台了财税、货币、投融资、区域和产业发展等方面的政策，这些政策将会为株洲的发展带来诸多利好。三是国家宏观战略布局调整带来的新机遇。株洲位于湖南“一部一带”战略定位的核心区，具备对接国家“三大战略”和承接沿海产业转移的区位、交通、科技、人才和资源等优势条件，这将给株洲带来巨大的发展潜力和市场空间。特别是株洲作为国家新型城镇化综合试点城市、国家自主创新示范区、国家老工业基地搬迁改造试点城市和国家两型社会建设综合配套改革试验区，必将获得更强的发展动力。

应对新挑战，把握新机遇，要求我们切实抓好项目攻坚，以项目建设强实力、增后劲；要求我们进一步深化改革，不断激发新动力，释放新红利；要求我们不断优化发展环境，为经济社会发展提供坚强保障。应对新常态，我们大有可为，必须坚定信心，勇毅笃行，主动作为，增创新优势，实现新发展，再铸新辉煌。

2015年政府工作的指导思想是：全面贯彻党的十八大、十八届三中、四中全会和中央、省委经济工作会议精神以及市委的决策部署，主动适应经济新常态，坚持稳中求进、好中求快的工作总基调，坚持以提高经济质量和效益为中心，紧扣“三个率先”的总目标，深入实施转型升级总战略，集中力量打好 “四大攻坚战”，扎实开展“项目攻坚年、改革深化年、环境优化年”活动，突出创新创业，大力发展民营经济，加强民生保障，注重底线思维，促进经济平稳健康较快发展和社会和谐稳定。

今年全市经济社会发展的主要预期目标是：GDP增长9.5%，力争增长10%；规模工业增加值增长11.5%；公共财政预算收入增长10%；全社会固定资产投资增长20%；社会消费品零售总额增长12.5%；居民消费价格涨幅控制在3%以内；新增城镇就业6.1万人；城乡居民收入分别增长10%、11%；万元GDP能耗下降3%以上。各项事业都有新的发展。

与此同时，我们将集中力量抓好：十大基础设施项目、十大农业产业化项目、十大工业项目、十大服务业项目、十大重点改革和“民生100”工程。（见附件）

三、2015年重点工作

围绕今年的指导思想和目标要求，我们将重点抓好以下六个方面的工作：

（一）坚持多措并举，着力促进经济平稳增长。致力稳投资、促消费、扩出口，推动全市经济平稳健康较快发展。

发挥投资的关键作用。扩大有效投资，优化政府投资，激活民间投资，加强招商引资，全力推进一批有利于壮大实体经济、有利于创造消费需求、有利于改善民生的重大项目，保持投资合理较快增长。重点实施“422”工程，即切实抓好40个重大项目、200个重点项目，带动完成2000亿元以上投资。建立健全项目建设推进机制，加强重点项目建设计划、融资计划、土地供应计划、征拆征收计划相互衔接、相互支撑。加强政银企合作，引导金融机构继续扩大信贷规模，支持企业通过上市、债券、票据、信托、基金、私募股权等多种方式扩大融资规模，为项目落地和建设提供坚强保障。积极鼓励和支持民间资本参与公共领域投资，在城镇基础设施、教育、医疗卫生等领域形成示范带动效应。进一步放低创业门槛，放活创业方式，掀起万众创新、大众创业新高潮。

发挥消费的基础作用。加强消费引导。根据消费需求，完善消费市场体系，充分开拓农村市场，发掘城市中等收入群体市场，重视高端消费品市场，满足群众多样化、个性化、便利化的消费需求，形成扩大消费的长效机制。培育消费热点。大力引导和促进汽车、旅游休闲、教育培训、文化创意、健康养老、家政服务等消费需求，拓展消费空间，提高生活品质。营造消费环境。强化通胀预期管理，加强粮、油、肉、菜等生活必需品市场监管，完善市场运行监测体系和生活必需品应急储备体系，稳定物价水平，保障市场平稳运行。推进商务执法体系建设。依法打击制售假冒伪劣产品、价格欺诈等不法行为，切实保护消费者合法权益。

发挥出口的支撑作用。加快“走出去”步伐，支持轨道交通、航空、硬质合金、陶瓷、服饰等企业开拓海外市场。支持有实力的企业到境外开发资源、承包工程、投资办厂，开展品牌、技术和生产线等并购，提高国际市场竞争力。壮大外贸主体队伍，着力培育重点外贸企业，力争年内业绩增长20%以上。支持外贸综合服务企业发展，为小微企业出口提供专业化服务。推动外贸转型升级，围绕建设轨道交通装备外贸基地和国家外贸转型升级示范基地，打造一批具有自主知识产权和品牌的出口产品。

（二）坚持改革开放，着力增创新的发展优势。大力开展“改革深化年”活动，以改革聚合正能量，激发新动力，释放新红利。

全面深化经济体制改革。深化投融资体制改革。加快完善政府投资管理配套办法，健全非经营性政府投资项目代建机制，推进政府投资项目分类管理，全方位、全过程规范政府投资行为。创新政府投融资平台管理体制，科学合理剥离政府与投融资公司债务，促进做大做强。鼓励社会资本参与公共领域项目建设，推行PPP、资产证券化、股权融资、金融租赁等模式。深化商事制度改革。积极探索“三证合一”登记制度，降低准入门槛，激发市场活力。深化金融体制改革。加快地方法人金融机构组建步伐，基本完成城区、县城农信社改革和村镇银行全覆盖。强化金融产品和服务创新，完善中小企业信用信息征信平台建设，有效缓解中小微企业融资难、融资贵问题。深化财税体制改革。加大预决算公开力度，不断完善全口径政府预算管理。加强专项资金绩效评价，提高财政支出绩效。继续抓好“营改增”工作。深化土地管理制度改革。积极稳妥推进土地承包经营权、农村集体建设用地使用权、宅基地确权颁证工作，加快土地交易平台建设，有序引导土地经营权流转，促进农业适度规模经营，通过土地“增减挂钩”推进城乡统筹发展。加快公共资源交易管理体制改革。组建统一的公共资源交易中心，构建公平公正公开透明的公共资源交易制度体系。

统筹推进其他领域改革。全面完成政府机构改革，稳步推进事业单位分类改革。抓好省直管县试点工作，稳妥推进乡镇区划调整，加快推进攸县撤县建市和株洲县撤县建区工作。加快推进在株央企和省属企业“三供一业”分离移交工作，加快完成市属国有企业改革。

积极扩大对外开放合作。构筑大通道。主动对接“一带一路”倡议，扩大“属地申报、口岸验放”和“属地申报、属地验放”适用范围。推进跨境电子商务应用，对接“湘欧快线”，促进互联互通，拓展发展新空间。搭建大平台。做好申报国家级经济开发区前期准备工作，加快推进湘赣开放合作试验区建设，争取长沙至深圳铁海联运在株洲设立运输节点。推进大招商。围绕“五城四基地”等重点产业开展专题招商活动，推进产业链和产业集群招商，强化园区招商、小分队招商和以商招商，引进央企国资、优质民资、高端外资。建立评估机制，注重税收贡献度、产业关联度和环境承载度，提高项目招商质量和效益。全年引进外资、内联引资均增长15%。

（三）坚持创新驱动，着力推进产业转型升级。大力实施创新驱动战略，开发新技术、培育新产业、创造新模式、打造新业态，推动经济提质增效升级。

发挥创新引领作用。启动国家自主创新示范区建设。围绕建成创新驱动发展引领区、科技体制改革先行区、军民融合创新示范区、中西部地区发展新的增长极，积极探索新机制、新模式。强化政策引导支持。全面落实自主创新示范区“6+4”政策，促进技术与市场融合、创新与产业对接。强化科技创新平台。新建大功率半导体器件国家重点实验室等一批企业研发平台，组建轨道交通自动化协同创新平台等一批科技创新公共服务平台。建立以企业为主体的技术创新体系。鼓励产学研合作，重点实施“万名人才计划”，依托园区、企业、高校和科研院所培育和引进创新人才，引进一批由院士领衔的科技创新团队。重点攻克一批关键技术，转化一批重大科技成果。力争高新技术产业产值突破1600亿元，增加值突破500亿元。

加速推动工业振兴。发展壮大优势产业。围绕“五城四基地”，做大龙头企业，提升产业配套能力，积极开拓国内外市场。重点围绕建设“中国动力谷”，以南北车合并重组、国家轨道交通装备产业集聚发展试点为契机，加快建设轨道交通科技城，推进轨道交通产业年内突破千亿大关。同时，加快推进航空城、汽车城等项目建设。加快培育新兴产业。围绕电子信息、新材料、新能源和食品医药四大新兴产业，重点加快IGBT应用产业化、新能源汽车、交通装备新材料、有色金属新材料、医药健康等领域发展，努力培育新的经济增长点。改造提升传统产业。大力推进有色冶金、化工、建材等工业技术改造。推进园区提档升级。坚持产城融合发展，以提高园区承载能力为突破口，继续推进园区“五个一批”工程，加快轨道交通科技城、航空城、服饰城、天易科技城等园区基础设施项目建设。突出发展工业地产，重点实施创新创业园区“146” 工程。积极推行专业化园区运营模式，完善园区公司运作机制，进一步释放园区发展活力。深入实施企业成长“十百千”工程，进一步强化对口服务、首问服务和代理服务。大力推进“个转企”、“小升规”工作。

促进服务业发展升级。大力发展生产性服务业。突出发展物流业，努力建设具有枢纽功能的物流体系，着力把交通区位优势转化为物流优势。大力发展信息服务、现代金融、中介服务、研发设计等服务业。大力提升传统服务业。统筹城乡流通体系建设，形成布局合理的城乡商业业态体系。大力实施流通产业“1511”培育工程，加快推进城铁株洲站综合体、电商产业园等重点项目建设，打造一批特色商贸和物流业集聚区。大力发展文化旅游业。发挥历史文化旅游、红色旅游、生态旅游和工业旅游的资源优势，做好整合文章，加快做大做强。以炎帝陵为龙头，整合炎陵、茶陵等地历史、文化、生态资源，促进生态文化旅游集聚发展。大力发展电力机车、服饰、陶瓷、航空等产业文化博览和产品会展业，举办好中国陶瓷艺术大展暨首届湖南（醴陵）陶瓷博览会，培育工业文化旅游新业态。

加快转变农业发展方式。启动农业产业升级“十百千”三年行动计划，加快发展现代农业。壮大农业经营主体。启动“万名职业农民培训三年计划”，加快形成以家庭承包农户为基础，专业大户、家庭农场、合作社组织、龙头企业为支撑，其他形式为补充的新型农业经营主体体系。调优农业产业结构。加强高标准农田建设，实施杂交稻高产示范工程、“种三产四”丰产工程、粮食高产工程，坚决制止耕地抛荒，确保粮食播种面积稳定在380万亩以上，总产180万吨以上。创新农业经营方式。加快农业由生产导向向消费导向转变，引入产业链、价值链等现代产业组织方式，加快一二三产业融合，做大做强特色农业、设施农业、加工农业、外向农业和“互联网”农业，发展创意农业、休闲农业。加快打造畜禽水产品加工、竹木加工、粮油加工、养殖业、饲料产业五个百亿产业。

（四）坚持新型城镇化，着力推进城乡统筹发展。抢抓国家新型城镇化综合试点城市机遇，继续深入打好城镇扩容提质攻坚战和县域崛起攻坚战，加快构建以人为核心、以产业为支撑、以改革为动力、以特色取胜的城镇化发展格局，促进城乡共同繁荣。

拓展城镇发展空间。坚持规划引领。实现城区控规全覆盖，市域村庄规划全覆盖。按照“一核三轴三带”城镇空间结构和“1349”城镇发展分区结构，构建新型城镇布局体系。加快城区扩容步伐。按照“北联、东拓、南进”的发展导向，加快建设云龙新城、东部新城和南部新区，着力构建“一体三极”的城区发展新格局。云龙新城重点发展文化旅游、职业教育、电子信息等产业，以低碳高增长发展模式，打造国家两型社会示范区。东部新城以莲株公路、服饰大道、机场大道和通用机场为支撑，重点发展航空城和服饰城，打造航空和服饰产业集群。南部新区以湘江大道和科技大道为支撑，沿湘江两岸重点发展天易科技城、渌口新区和南洲新区等组团片区，打造以产业园区为基础、生态为特色、产城一体的宜居宜业生态新区。

加强城乡基础设施建设。探索建立多元化、可持续的城镇基础设施建设投融资机制，努力建好“五网”。一是交通网。加强对外联通，加快长株潭城际铁路及配套工程、莲株公路、通用机场及株洲湘江航电枢纽二线船闸建设，打通洞株路、铜霞路、西站南路，加快与长沙、湘潭对接。完善市区交通，加快中环大道贯通工程、湘江大道、湘江六桥、湘江七桥建设，加快实施城市交通堵点畅通工程。完善市域交通网络，加快启动东城大道、渌枫大道等项目建设。年内建成国省干线公路100公里、新开工58公里、建成农村公路149公里。二是水利网。着力推进“一江两水”治理与中小河流项目建设，统筹推进县域供水和农村水利设施建设，抓好大中型灌区续建配套与节水改造、中小型病险水库除险加固，建设“五小”水利工程1250处。加强第二水源建设论证工作。三是能源网。加快推进天然气市域全覆盖工程，实现2016年县县通天然气。抓好农网改造工程。积极推进风力发电场建设。大力推进农村大中型沼气、太阳能、小水电等代燃节能工程。四是信息网。着力推进3G网络基本覆盖城乡、4G网络初步规模商用，无线局域网覆盖公共热点领域。以信息化应用工程建设为抓手，推动智慧株洲建设。五是城镇配套设施网。改造和完善城市排水防涝管网。建设覆盖城乡的垃圾收运和处理系统。加快推进城市棚户区、城中村、背街小巷和农贸市场改造。推动有轨电车项目建设，抓好公交站点、公共停车场建设。

加速推进县域崛起。以全面建成小康社会为总揽，坚持“五位一体、四化同步”的思路，提升县域综合实力。增强县域产业支撑。以产业园区为载体，发展壮大农产品加工、陶瓷、文化旅游等区域优势产业，大力承接产业转移，培育县域产业新支柱。不断提升园区规模和功能，培育县域经济增长极。加快重点城镇建设。按照“扩大财权、下放事权、增加地权”的要求，推进扩权强镇，打造一批产业特色明显、综合实力较强的工业强镇、商贸重镇、文化古镇、旅游名镇、省际边界要镇。推进美丽乡村建设。大力实施“213”示范工程，推进新农村建设向示范片、示范圈、示范带跨越，打造美丽乡村、完美社区。深入实施乡村清洁工程，推进农村危房改造、马路市场取缔、空心村整治，切实改善农村人居环境。加大扶贫攻坚力度。实施精准扶贫，促进茶陵、炎陵等地贫困人口加快脱贫致富步伐，全年减贫5万人。

创新城乡综合管理机制。加快市政管理体制改革，逐步将城镇设施维护、园林绿化养护、灯饰管养、渣土清运等领域向市场有序放开，进一步完善市场化运营模式。推动数字城管、智能交通系统升级，启动城市公共信息资源数据中心等项目建设，进一步提升城市管理网格化、数字化、精细化和标准化水平。抓好创建活动开展，加快国家环保模范城市、国家公交都市创建步伐，支持县级城区开展创建活动，不断提升城镇品质品位。改革完善户籍管理制度，探索建立政府、企业和个人成本分担机制，统筹教育、卫生、文体、养老等设施规划布局，增强城镇公共服务能力，加快推进农业转移人口市民化进程。

（五）坚持两型引领，着力建设生态宜居家园。以两型社会建设为引领，坚持和强化大环保格局，注重培育两型文化，持续打好两型攻坚战，全面完成试验区第二阶段任务。

全力开展污染防治。狠抓水污染治理。大力实施好湘江保护与治理省政府“一号重点工程”，积极稳妥推进清水塘老工业基地搬迁改造。全面完成“三个基本”行动计划，扎实做好“一江四港”和湘江河东城区段综合治理，重点加强工业废水、生活污水、养殖污染等源头控制。狠抓大气污染治理。强化工业废气和机动车尾气污染防治，重点完成城区煤改天然气等35个大气治理项目，淘汰黄标车1.2万辆，实现县城PM2.5监测全覆盖，力争全年空气质量优良率提高5个百分点。狠抓土壤污染治理。全力推进重金属污染治理项目建设，抓好污染耕地修复和农作物种植结构调整。做好矿区生态环境综合治理工作。

切实保护和建设生态环境。巩固和扩大森林城市建设成果，实施城镇绿荫行动，加强生态功能区、风景名胜区、森林公园、湿地等重点区域的保护与管理，全年植树造林面积20万亩以上。切实加强生态主体功能区保护和建设。大力推进全国水生态文明城市试点工作。建立两型技术及产品发展和应用、领导干部自然资源资产离任审计等制度，建立生态补偿机制、联动执法机制，完善生态文明考核评价体系。

突出推进资源节约集约利用。坚持问题导向，加快改造或淘汰高能耗、高排放、高污染企业，全面推进重点企业、公共机构和设施节能改造。优化能源结构，推广新能源和清洁能源，加快光伏发电、风力发电、余热发电、分布式能源站等项目建设。加强建筑节能工作。建立以土地利用效益为导向的土地管理制度，清理、盘活低效建设用地，提高土地投入强度和产出水平。全面推广节水技术和产品，大力推广节材新工艺、新技术，开展垃圾分类处理和废弃物资源化利用工作，发展节能环保产业。

全面推进集成示范创建。天易、云龙等片区全面推广可实施的两型标准、技术和发展模式，力争率先建成两型集成示范区。基本建成神农城、职教科技园2个省级综合示范片区和8个市级综合示范片区。重点在全市各类机关、企事业单位、社区全面开展两型示范创建，带动14个类别创建全面推进。及时总结、提升两型标准、创建规程、经验模式，发挥好示范引领作用。

（六）坚持民生优先，着力增进人民群众福祉。按照“建机制、补短板、兜底线”的要求，以实施“民生100”工程为抓手，办好一批让老百姓看得见、摸得着、感受得到的民生实事，让发展成果更多更公平地惠及全市人民。

促进创业就业。以富民为目标，出台鼓励全民创新创业政策，设立创业扶持基金，以创业带动就业，让想创业的人创新业，正创业的人创好业，善创业的人创大业。加强创业培训，建设农民就业创业基地。力争全年实现新增农村劳动力转移就业1.6万人，零就业家庭动态援助率100%，城镇登记失业率低于4.5%。完善最低工资标准、工资指导线、工资集体协商等制度，着力构建和谐劳动关系。

加强社会保障。实施社会保险全覆盖工程，启动全民参保登记工作，做到应保尽保。推进机关事业单位养老保险制度改革。推进城乡居民基本养老保险制度并轨实施。推进医保城乡统筹，完善医疗保险、生育保险市级统筹，深化医保支付制度改革。完善被征地农民社会保障政策，完善城乡居民大病保险制度，完善社会救助体系，保障特殊困难群众基本生活。解决27.21万农村人口饮水不安全问题。健全住房保障和供应体系，推进公共租赁住房和廉租住房并轨改革，实施棚户区改造1.8万户以上，开工建设公租房（廉租房）1万套以上，规范保障性住房建设、分配和管理工作。加快完善社会养老服务体系。保障妇女儿童合法权益，关心和支持残疾人事业，加快发展公益慈善事业。

繁荣社会事业。围绕建设教育强市，全面改善农村薄弱学校的办学条件，年内实现农村教学点数字资源全覆盖。启动学前教育、职业教育两个三年行动计划，实现公办幼儿园覆盖各乡镇。加快职教科技园建设。加快市特殊学校新校区建设。深化公立医院改革，完善现代医院管理制度，着力构建分级诊疗格局。鼓励社会力量办医，积极探索多方参与的市场办医模式。继续实施临床医学重点专科建设三年行动计划，进一步完善公共卫生服务体系，促进基本公共卫生服务均等化。大力创建国家公共文化服务示范城市，加快推进公共文化设施建设和公共文化产品供给。加强文物保护和开发利用。深入开展全民健身工程，发展体育产业，办好全市第五届大众体育运动会。稳妥实施“单独两孩”政策。推进统计制度改革和信息化建设。继续做好老龄、民族宗教、广播电视、文化执法、对台、档案、市志、气象、志愿者服务等工作。

创新社会治理。以社会治理网格化为抓手，提高预防和处置突发事件能力，创新社会矛盾纠纷化解机制。深入推进“平安株洲”建设，加快“智慧城市”电子眼及联网项目建设，强化突发敏感舆情和公共应急管理。健全安全生产工作机制和责任体系，确保全市安全生产形势平稳可控。加强食品药品安全监管，确保“舌尖上的安全”。统筹推进国防动员、民兵预备役、武警、应急动员、人防及军事设施保护等工作。做好征兵及双拥共建工作，推动军民融合发展。

四、加强政府自身建设

应对经济发展新常态，关键要加强政府自身建设。要坚持问题导向、市场导向、需求导向，推进依法行政，着力简政放权，加强管理创新，改进工作作风，提高治理能力。

（一）切实推进依法行政。健全依法决策机制，严格落实公众参与、专家论证、风险评估、合法性审查、集体讨论决定等重大行政决策程序，推进政府决策科学化、民主化和法治化。深化行政执法体制改革，推行跨部门综合执法，健全行政裁量权基准制度，全面落实行政执法责任制，依法惩处各类违法行为，切实维护人民群众的合法权益。认真落实人大及其常委会各项决议决定，积极主动接受人大及其常委会的法律监督、工作监督和政协的民主监督。切实加强同人大代表和政协委员的联系，认真办理好建议提案。广泛听取各民主党派、工商联、无党派人士和人民团体的意见，重视司法、舆论、公众监督，提高依法行政水平。

（二）切实转变政府职能。努力把该放的权放开放到位，把该管的事管住管好。继续做好行政审批事项的承接落实和取消下放工作，全面出台“权力清单”、“责任清单”、“负面清单”三个清单，做到法无授权不可为，法定职责必须为，保障公民和法人法无禁止即可为。对保留的审批事项，不断创新审批方式，全面优化审批流程，强化事中、事后监管。积极培育和规范中介机构，服务经济社会发展，优化部门职责体系，最大限度地整合分散在不同部门相同或相近的职责，努力解决政府职能缺位、越位和错位问题。

（三）切实创新政府管理。更多地运用市场化、社会化、信息化的方式提高行政效率。扩大政府向社会力量购买公共服务试点。充分发挥社会信用体系规范市场秩序、社会秩序的作用，推进信用体系建设。全面推行政务公开，加强电子政务平台建设，创新和完善政务公开制度体系，让权力在阳光下运行。切实提高政府部门办事效率，坚决整治“为官不为”，以问题导向、目标管理倒逼效能提升。

（四）切实改进工作作风。巩固和发展党的群众路线教育实践活动成果，持之以恒反“四风”，驰而不息肃政纪。严格落实党风廉政建设责任制，强化对重要岗位、关键环节的监督制约。加大监察、审计的监督力度，严格执行廉洁从政各项规定。增强改革创新意识，鼓励敢闯敢试，强化责任担当，严格执行工作责任制、项目负责制、行政问责制。牢固树立群众观念，感同身受群众困难，通过一件件实事的办理、一件件难事的解决，赢得人民群众更多的信任和更大的支持。

科学编制“十三五”规划是今年的一项重大任务。我们将立足市情，深入研究经济新常态下的新情况，准确把握重大机遇和挑战，研究提出发展目标和任务，精心策划一批重大项目，描绘好株洲发展的美好蓝图。

各位代表！时代赋予重任，实干成就梦想。让我们在省委、省政府和市委的坚强领导下，万众一心，团结奋斗，以更大的担当、更实的作风，攻坚克难，锐意进取，为在新常态下打造好株洲发展升级版而努力奋斗！
